# stAA: adversarial graph autoencoder for spatial clustering task of spatially resolved transcriptomics

**DOI:** 10.1093/bib/bbad500

**Published:** 2024-01-06

**Authors:** Zhaoyu Fang, Teng Liu, Ruiqing Zheng, Jin A, Mingzhu Yin, Min Li

**Affiliations:** School of Computer Science and Engineering, Central South University, Changsha, Hunan 410083, China; Clinical Research Center (CRC), Medical Pathology Center (MPC), Cancer Early Detection and Treatment Center (CEDTC), Chongqing University Three Gorges Hospital, Chongqing University, Chongqing 404031, China; Translational Medicine Research Center (TMRC), School of Medicine, Chongqing University, Chongqing 401331, China; School of Computer Science and Engineering, Central South University, Changsha, Hunan 410083, China; School of Computer Science and Engineering, Central South University, Changsha, Hunan 410083, China; Clinical Research Center (CRC), Medical Pathology Center (MPC), Cancer Early Detection and Treatment Center (CEDTC), Chongqing University Three Gorges Hospital, Chongqing University, Chongqing 404031, China; Translational Medicine Research Center (TMRC), School of Medicine, Chongqing University, Chongqing 401331, China; School of Computer Science and Engineering, Central South University, Changsha, Hunan 410083, China

**Keywords:** spatial transcriptomics, spatial domain, graph neural network, adversarial learning, graph autoencoder

## Abstract

With the development of spatially resolved transcriptomics technologies, it is now possible to explore the gene expression profiles of single cells while preserving their spatial context. Spatial clustering plays a key role in spatial transcriptome data analysis. In the past 2 years, several graph neural network-based methods have emerged, which significantly improved the accuracy of spatial clustering. However, accurately identifying the boundaries of spatial domains remains a challenging task. In this article, we propose stAA, an adversarial variational graph autoencoder, to identify spatial domain. stAA generates cell embedding by leveraging gene expression and spatial information using graph neural networks and enforces the distribution of cell embeddings to a prior distribution through Wasserstein distance. The adversarial training process can make cell embeddings better capture spatial domain information and more robust. Moreover, stAA incorporates global graph information into cell embeddings using labels generated by pre-clustering. Our experimental results show that stAA outperforms the state-of-the-art methods and achieves better clustering results across different profiling platforms and various resolutions. We also conducted numerous biological analyses and found that stAA can identify fine-grained structures in tissues, recognize different functional subtypes within tumors and accurately identify developmental trajectories.

## INTRODUCTION

In recent years, single-cell RNA sequencing (scRNA-seq) technologies have made significant progress in understanding cellular subpopulations and individual cell biology [1]. However, while scRNA-seq can detect gene expression profiles, it lacks the ability to retain spatial positional information. In contrast, spatial transcriptomics protocols enable the detection of both gene expression profiles and spatial positions [2], which enhances our understanding of molecular communication and tissue architecture [3]. Various spatial transcriptomics methodologies have been developed, including seqFISH+ [4], MERFISH [5], Slide-seqV2 [6], 10X Visium [7], Stereo-seq [8] and 10X Xenium [9]. These spatial sequencing technologies enable the identification of spatial domains and further analysis of tissue substructures using spatial location information.

Accurate spatial domains annotation is one of the key steps of spatial transcriptome data analysis, including spatial domain function, spatial organization reconstruction, cell-to-cell communication, spatial trajectory inference, etc. To this end, a number of clustering approaches have been developed for domain identification, which are mainly cast into three categories: traditional, probabilistic and deep learning methods. Typically, the traditional techniques are *K*-means, Louvain and Leiden algorithms, which are often used in Scanpy [10] or Seurat [11] packages to constitute the integrated analysis workflow. The foundation of the probabilistic clustering methods is the Markov random field. For example, Giotto [12] employs a hidden Markov random field to recognize spatial domain by comparing the inherent gene expression patterns of adjacent cells. Based on a fully Bayesian statistical method, BayesSpace [13] encourages neighboring cells to belong to the same group via a pre-defined spatial prior. Moreover, SC-MEB [14] improves the hidden Markov random field by optimizing the smoothing parameter with an empirical Bayes model. However, these probabilistic techniques do not effectively utilize the available spatial coordinates. More recently, an increasing number of spatial clustering methods related to deep learning have been proposed, which typically leverage the power of graph neural networks (GNNs).

GNNs are found to be powerful and versatile in combining gene expression profiles and spatial context to perform spatial clustering task. For example, stLearn [15] integrates gene expression normalization, spatial location and morphological adjustment to search graph-based clusters. SEDR [16] uses two autoencoders to address the correlative information in spatial transcriptome. A deep autoencoder handles the gene expression matrix and a variational graph autoencoder disposes of the spatial locations. SpaGCN [17] employs an undirected weighted graph to represent the dependency of spatial data, wherein the spatial position, histological image and gene expression participate in graph construction. STAGATE [18] adopts a graph attention autoencoder to distinguish spatial regions, with an attention mechanism discerning the similarity of contiguous cells and a cell type-aware module responsible for pre-clustering. Furthermore, a graph convolutional network-enabled unsupervised cell clustering approach (CCST) is formulated in [19], and the deep graph infomax (DGI) module is employed to maximize mutual information. DeepST [20] leverages a denoising autoencoder and a GNN autoencoder to jointly derive the latent embedding of augmented spatial transcriptomic data. These existing spatial clustering methods often encounter the problem of learning degenerate identity mappings, where the latent embedding space does not have meaningful structure. In addition, GNN can be prone to overfitting [21], which hinders the accurate identification of spatial domain boundaries.

In this article, we propose stAA, a novel Adversarially regularized variational graph Autoencoder for spatial clustering task. We use the variational graph autoencoder model to minimize the reconstruction errors of the spatial graph and introduce Wasserstein generative adversarial network (WGAN) [22] to produce a latent embedding with a prior distribution. By utilizing an adversarial learning mechanism, our model stAA increases the distance between latent embeddings of different cells, which leads to a higher variance of the embeddings and ultimately better reconstruction. Moreover, we construct a classifier that enables the latent embedding to learn global graph information provided by the pre-clustering cluster labels. As a result, stAA generates robust and meaningful latent embeddings that are better suited for downstream clustering tasks.

We evaluate stAA on multiple datasets of human and mouse tissues, such as 10X Visium, Slide-seqV2, Stereo-seq and STARmap. The relevant tissues include human brain, human breast cancer, mouse hippocampus, mouse olfactory bulb and mouse embryo. The experimental results demonstrate that stAA is superior to the six state-of-the-art spatial clustering methods, including SpaGCN [17], conST [23], DeepST [20], CCST [19], STAGATE [18] and GraphST [24]. In general, stAA is proven to possess great power in recognizing spatial domains and is scalable for additional spatial transcriptome datasets.

## METHODS

### The overview of the stAA

#### Variational graph autoencoder

To fully leverage both spatial location information and gene expression data, stAA first utilizes the spatial location information to construct a graph $G$ (see Supplementary Note 1 and Supplementary Note 1 for details), whose adjacency matrix is represented as $A$. In the graph $G$, each node represents a spot, which can be a single cell, multiple cells or even subcellular. For the adjacency matrix $A$, Aij = 1 indicates that the spot $i$ and $j$ have a close relationship, while Aij = 0 indicates that the spatial distance between spot $i$ and $j$ is far apart. $X$ is the gene expression matrix with each row representing the gene expression profile of the corresponding spot. The objective of a graph neural network is to map the graph $G$ into a latent space $\mathrm{Z}$ while retaining the topological structure and content information, which is the encoding process [25]. The decoding process is to reconstruct the adjacency matrix ${A}^{\prime }$ using the latent matrix $Z$ and ensure ${A}^{\prime }$ to be close to $A$.

To represent both the feature matrix $X$ and the adjacency matrix $A$, the generator ${G}_W$ uses the GNN to perform low-dimensional representation. In the first layer of GNN, a lower-dimensional feature matrix $\overset{\sim }{X}$ is computed as follows:


(1) \begin{equation*} \overset{\sim }{X}={f}_{\mathrm{Relu}}\left(X,A|{W}_{G1}\right)=\mathrm{Relu}\left( AX{W}_{G1}\right) \end{equation*}

where $\mathrm{Relu}(t)=\max \left(0,t\right)$ is the rectified linear activation function and ${W}_{G1}$ is the weight matrix of the first layer GNN named G_1_.

In the second layer of GNN named G_2_, the mean and variance vectors of the feature matrix $\overset{\sim }{X}$ are determined by training the weight matrices ${W}_{G2}$ and ${W}_{G{2}^{\prime }}$ as


(2) \begin{equation*} {\displaystyle \begin{array}{c}\mu \kern0.3em =\kern0.3em {f}_{\mathrm{linear}}\left(\overset{\sim }{X},A|{W}_{G2}\right)=A\overset{\sim }{X}{W}_{G2}\\{}\log{\sigma}^2=\kern0.3em {f}_{\mathrm{linear}}\left(\overset{\sim }{X},A|{W}_{G{2}^{\prime }}\right)=A\overset{\sim }{X}{W}_{G{2}^{\prime }}\end{array}} \end{equation*}

where $\mu$ is the mean vector and ${\sigma}^2$ is the variance vector. It should be noted that GNN could have multiple hidden layers; we only exhibit one layer for comprehension. Finally, the latent embedding $Z$ is calculated via a reparameterization trick [26] by integrating the mean and variance vector:


(3) \begin{equation*} Z=\mu +\sigma \odot \varepsilon \end{equation*}

where $\varepsilon \sim \mathcal{N}\left(0,1\right)$ is the standard normal distribution and $\odot$ is the Hadamard point-wise multiplication operator.

The decoder in Variational Graph Autoencoder (VGAE) module aims to reconstruct the adjacency matrix $A$ in the spatial clustering task. The incoming and prediction distributions of the decoder are described as $q\left(\left.Z\right|X,A\right)$ and $p\left(\left.{A}^{\prime}\right|Z\right)$. Concretely, a prediction layer is trained for the spatial neighbor graph according to the latent embedding:


(4) \begin{equation*} p\left(\left.{A}^{\prime}\right|Z\right)=\mathrm{sigmoid}\left(Z{Z}^T\right) \end{equation*}

where $\mathrm{sigmoid}(t)=\frac{1}{1+{\mathrm{e}}^t}$ is the logistic sigmoid function. The high similarity of the incoming and prediction distributions illuminates that the latent representation could reserve the feature and spatial information. The binary cross-entropy loss function is reasonable to consist of the reconstruction loss and variational lower bound as follows:


(5) \begin{equation*} {L}_1={E}_{q\left(\mathrm{Z}|\mathrm{X},\mathrm{A}\right)}\left[\log p\left({A}^{\prime }|Z\right)\right]- KL\left(q\left(Z|X,A\right)\Big\Vert p(Z)\right) \end{equation*}

wherein $KL\left(q\Big\Vert p\right)$ indicates the Kullback–Leibler divergence between two probability distributions. In this manuscript, an adversarial model is established to further improve the quality of the latent embedding.

To improve the ability to identify spatial domain boundaries, we add a classifier in the VGAE model to further improve the quality of the latent embedding. Firstly, we segment the gene expression profiles $X$ using a traditional method (mclust [27] or Louvain [28]) to obtain the initial labels $y$. These initial labels $y$ guide the latent embedding $Z$ to better capture the structure of the gene expression profiles $X$. The classifier ${C}_{\varphi }$ takes the latent embedding $Z$ as input and outputs the predicted label. The following cross-entropy (CE) loss [29] is used to train this classifier:


(6) \begin{equation*} {L}_2=y\log \left({C}_{\varphi }(Z)\right)+\left(1-y\right)\log \left(1-{C}_{\varphi }(Z)\right) \end{equation*}

This allows the latent embedding $Z$ to incorporate global information from initial labels $y$, thereby improving stAA’s ability to capture biological information of the gene expression profiles $X$.

#### Adversarial training model

In practice, the VGAE module usually learn degenerate identity mappings where the latent embedding space has no structure at all, which tends to result in poor performance when processing sparse and noisy data [30]. In this article, we address this issue by adding an adversarial training model. The common generative adversarial network (GAN) has defects of model collapse and parameters obscure [22]. Hence, a WGAN is used to address these disadvantages and enhance the latent representations. The adversarial learning model depends on a standard multi-layer perceptron to force the latent distribution ${Q}_g\left(z|A,X\right)$ into the target distribution ${P}_r\sim \mathcal{N}\left(0,I\right)$. Here, we use Wasserstein regularizer by a multi-layer perceptron with two hidden layers:


(7) \begin{equation*} {\mathfrak{R}}_{\theta }(Z)={W}_{R3}\sigma \left({W}_{R2}\left(\sigma \left({W}_{R1}Z\right)\right)\right) \end{equation*}

where ${W}_{R1}$ and ${W}_{R2}$ are weights in the hidden layers, while ${W}_{R3}$ is the parameter in the output layer. $\sigma$ represents the activation function, and we use the sigmoid function.

**Table 1:** Summary of all datasets in this study

Platforms	Organisms	Tissues	Slices	# Spots	# Genes
10X Visium	Human	Dorsolateral pre-frontal cortex (DLPFC)	151507 151508 151509 151510 151669 151670 151671 151672 151673 151674 151675 151675	4226 4384 4789 4634 3661 3498 4110 4015 3639 3673 3592 3460	20 494 20 083 20 732 20 475 20 583 20 338 21 037 10 725 21 267 21 897 20 783 20 806
		Breast cancer	Section_1	3798	36 601
Slide-seqV2	Mouse	Hippocampus	Puck_200115_08	41 786	4000
	Mouse	Olfactory bulb	Puck_200127_15	20 139	11 750
Stereo-seq	Mouse	Organogenesis	E9.5_E1S1 E9.5_E2S1 E9.5_E2S2 E9.5_E2S3 E9.5_E2S4	5913 5292 4356 5059 5797	25 568 23 756 24 107 24 238 23 398
STARmap	Mouse	Visual cortex	X	1207	1020

**Table 2:** The ablation results of stAA on the human DLPFC dataset

Samples Methods	Slice 151674	Mean	Median
All	0.60	0.56	0.57
w/o Classifier	0.55	0.52	0.53
w/o WGAN	0.53	0.51	0.51
w/o WGAN and classifier	0.52	0.50	0.51

The first column shows the ARI values of slice 151674. The second and third columns display the ARI values computed based on 12 samples.

The latent space acts as a generator and the multi-layer perceptron plays as a Wasserstein regularizer ${\mathfrak{R}}_{\theta }$. The goal of this multi-layer perceptron is to minimize the 1 − Wasserstein distance between ${Q}_g$ and ${P}_r$ [22]. The relevant loss of this Wasserstein distance (WD loss) is described as follows:


(8) \begin{equation*} {L}_3=-{W}_1\left({Q}_g,{P}_r\right)=-{E}_{r-{P}_r}\left[{\mathfrak{R}}_{\theta }(r)\right]+{E}_{z\sim{Q}_g}\left[{\mathfrak{R}}_{\theta }(z)\right] \end{equation*}

where ${W}_1$ is the 1 − Wasserstein distance. Finally, the generator in the proposed adversarial variational graph autoencoder is trained by combining the above three loss functions: the ${L}_1$ loss for construction, the ${L}_2$ loss for pre-clustering and the WD loss ${L}_3$ for adversarial training:


(9) \begin{equation*} L=f\left({L}_1,{L}_2,{L}_3\right)=\underset{w,\varphi }{\min}\underset{\theta }{\max}\left\{\lambda{L}_1+\left(1-\lambda \right){L}_2-{L}_3\right\} \end{equation*}

where $\lambda$ is a hyperparameter that adjusts the weight of reconstruction loss ${L}_1$ and classification loss ${L}_2$. The min and max in the loss function formulation represent adversarial optimization process between the generator and Wasserstein regularizer. The objective is a minimax game, where the generator aims to minimize the loss while the Wasserstein regularizer aims to maximize it. The min term pertains to the generator trying to minimize the discrepancy between the latent distribution and target distribution by reconstructing the adjacency matrix. On the other hand, the max term corresponds to the regularizer attempting to maximize the distance between the latent distribution and target distribution. This adversarial interplay leads to a balance, with both the generator and regularizer improving iteratively in response to each other’s performance. The latent embeddings generated using the learned encoder from Eq. (8) are utilized to identify spatial domains. These embeddings not only preserve the topological and content information of the original spatial graph but they also match the target distributions. As a result, the distance between latent embeddings of different samples increases, leading to higher variance of the latent embeddings and better reconstruction [30, 31]. Consequently, the learned embeddings become more meaningful and robust, leading to improved accuracy in downstream tasks.

**Figure 1:**
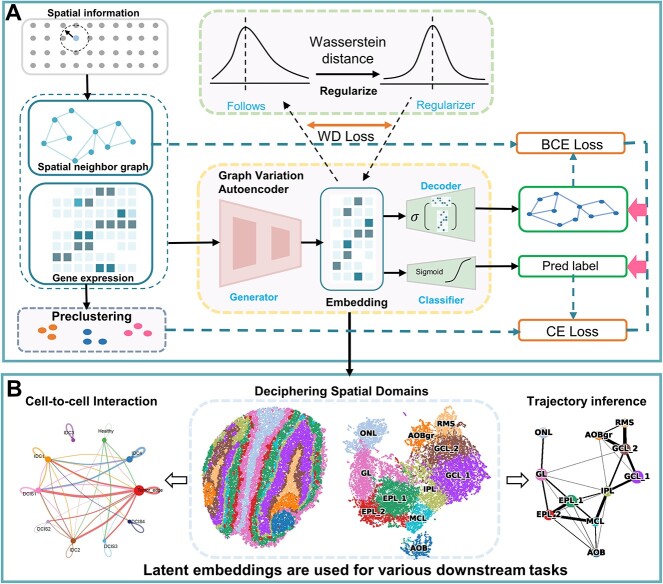
Overview of stAA. (**A**) A variational graph autoencoder model is applied to generate the latent embedding based on gene expression profiles and spatial information. The gene expression data is partitioned into diverse initial clustering according to a traditional method. A classifier enables the latent embedding to capture global graph information derived from the pre-clustering group labels. An adversarial training scheme is used to improve the quality of the latent embedding. The Wasserstein distance replaces a discriminator to differentiate between two probability distributions. (**B**) The generated latent embeddings are clustered using louvain or mclust. The resulting clustering can be used for various downstream analyses, including trajectory inference and cell-to-cell communication analysis.

Based on this embedding, the spatial domains are detected by the downstream clustering algorithms (we use mclust [27] or Louvain [28] in this paper). Hyperparameter settings can be found in Supplementary Note 2. The workflow of stAA is shown in Figure 1.

## RESULTS

### Datasets

To effectively evaluate the performance of stAA, we use datasets from four different platforms (10X Visium data, Slide-seq V2, Stereo-seq, STARmap) with varying sequencing depths (Table 1). The first dataset focuses on the human dorsolateral pre-frontal cortex (DLPFC) area, using 10X Visium and consisting of 12 samples [32]. This DLPFC data has been a standard dataset that each clustering algorithm would be verified on it first. The second and third datasets are based on Slide-seqV2 technology, focusing on the mouse hippocampus [33] and mouse olfactory bulb [18, 34], respectively. The fourth dataset is based on Stereo-seq, from the Mouse Organogenesis Spatiotemporal Transcriptome Atlas (MOSTA) database [35]. The fifth dataset is also provided by 10X Visium, derived from human breast cancer, with high heterogeneity and a complex microenvironment [16]. Finally, we use a mouse visual cortex dataset sequenced by STARmap [36] (see Supplementary Note 3 for details).

### Evaluation metrics

The most intuitive way to quantify the clustering performance is by justifying the differences between two clustering vectors. The evaluation criteria used depend on whether the true label of the spatial dataset is known or not. When the true label is known, the adjusted Rand index (ARI) is considered the estimation standard in this paper [25]. The ARI score is calculated based on the agreement between the true and predicted labels, taking into account chance agreement. This score provides a measure of the similarity between the true and predicted labels, and it ranges from −1 to 1, with higher values indicating better clustering accuracy.

When the ground truth of spatial transcriptomic data is not known, the Silhouette Coefficient (SC) score [27] and Davies–Bouldin (DB) index [28] are utilized to estimate the clustering performance. The SC value is determined by the mean intra-cluster distance and mean nearest-cluster distance [37]. The DB index is decided as the average similarity measure of each cluster with its most similar cluster. A bigger SC score and a smaller DB index indicate a better clustering performance.

### Comparison of stAA on human DLPFC dataset with SOTA spatial clustering algorithms

Firstly, we demonstrate that stAA can improve the accuracy and robustness of spatial clustering on the human DLPFC dataset. There are 12 samples in this dataset, and each sample has manual annotation. Four samples (151669, 151670, 151671 and 151672) have five clusters, while the remaining eight samples have seven clusters. We compared stAA with existing state-of-the-art methods including SpaGCN (16), conST (30), DeepST (19), CCST (18), STAGATE (17) and GraphST (31). We run the 7 methods on each sample 10 times with different random seeds.

**Figure 2:**
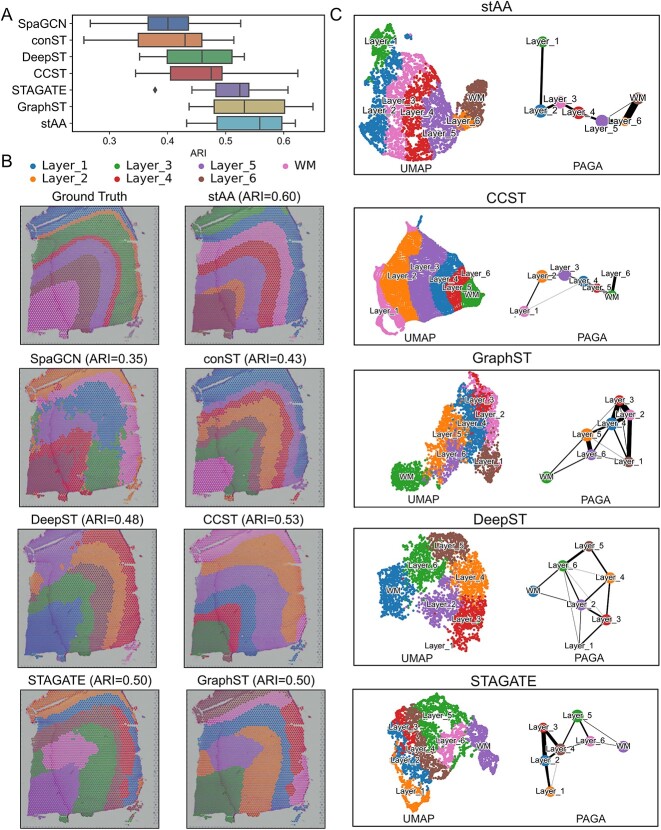
Comparison of stAA and other methods on the human DLPFC dataset. (**A**) The boxplots of average ARI values of seven approaches (SpaGCN, conST, DeepST, CCST, STAGATE, GraphST) for 12 samples. Each method is running 10 times to eliminate the random effects. (**B**) Ground truth for sample 151674, which has seven domains: six cortex layers and one white matter (WM). Spatial domain identification results for sample 151674 using stAA, SpaGCN, conST, DeepST, CCST, STAGATE and GraphST. (**C**) Visualization of UMAP and PAGA graphs generated by stAA, DeepST, CCST, STAGATE and GraphST, using their respective embeddings and clustering labels for sample 151674.

Figure 2A displays the average ARI [38] value of each technique for 12 samples. It is obvious that stAA has the highest average ARI, which is close to 0.6. The variance of each method is shown in Supplementary Figure 1. A lower variance indicates higher stability. This clearly demonstrates that stAA is the most robust. The conST and CCST have the worst performance. The variances in the remaining four methods (SpaGCN, DeepST, STAGATE and GraphST) are close. We show the results for a single sample (151674) in Figure 2B and C. The ground truth of sample 151674 is given in Figure 2B. stAA achieved the highest ARI of 0.60, which identifies relatively accurate spatial domain structure. This is followed by CCST with a score of 0.53. The ARI values in STAGATE and GraphST are the same, while those in SpaGCN, conST and DeepST are lower than 0.5 for this sample.

The embedding generated by stAA with Wasserstein distance regularization reveals clear spatial differentiation trajectories. In Figure 2C, the UMAP plot of stAA and six comparison approaches shows the generated embeddings and the trajectory inference results using PAGA [39]. It is apparent that each layer has its distinctive region in the stAA’s UMAP plot. The PAGA graph displays a linear trajectory from layer 1 to layer 6 and then to the white matter, with the similarity between adjacent layers. Although DeepST separated each layer of cells clearly, its inferred trajectories were not linear. CCST accurately inferred the linear trajectory of sample 151674, but there was a small group of layer 4 cells adjacent to layer 2 cells. STAGATE and GraphST methods show varying layers of overlap and misclassification between layers.

The detailed ARI values of seven methods for each sample are exhibited in Supplementary Figure 2. By comparing these ARI values, stAA achieves the highest score in eight samples. Among the six comparison methods, except for CCST, which is based on DGI, the other five methods, including stAA, are based on graph autoencoder. Overall, stAA achieves the best clustering performance, indicating that the WGAN and classifier modules added to the cell embedding in stAA are effective.

To further demonstrate the importance of the WGAN and classifier modules, we conducted ablation experiments on this dataset. The elaborate ARI scores are given in Table 2. These results prove the WGAN and classifier modules are both beneficial to the latent embedding generation. It can be discerned that the WGAN has the most significant influence on the clustering performance. This model can make the learned representations from VGAE more meaningful and informative. The classifier model provides initial guidance for the latent space, and thus the quality of latent embedding could also be improved. stAA achieves the best performance when these two models are used jointly, as displayed in Table 2. These ablation results can verify the necessity and effectiveness of the stAA framework.

To explore the impact of the number of hidden layers in GNN on the accuracy of spatial domain clustering, we conducted comparison experiments. While maintaining constant values for other model parameters, we employed one-layer, two-layer and three-layer GNN architectures separately. Supplementary Figure 3 displays ARI box plots for each scenario, revealing optimal performance when utilizing a single hidden layer.

### stAA can identify fine-grained structures of mouse hippocampus and mouse olfactory bulb tissues without ground truth

In this section, we compare the proposed stAA with two superior baselines, STAGATE [18] and GraphST [24], which were used as benchmarking methods for human DLPFC datasets.

**Figure 3:**
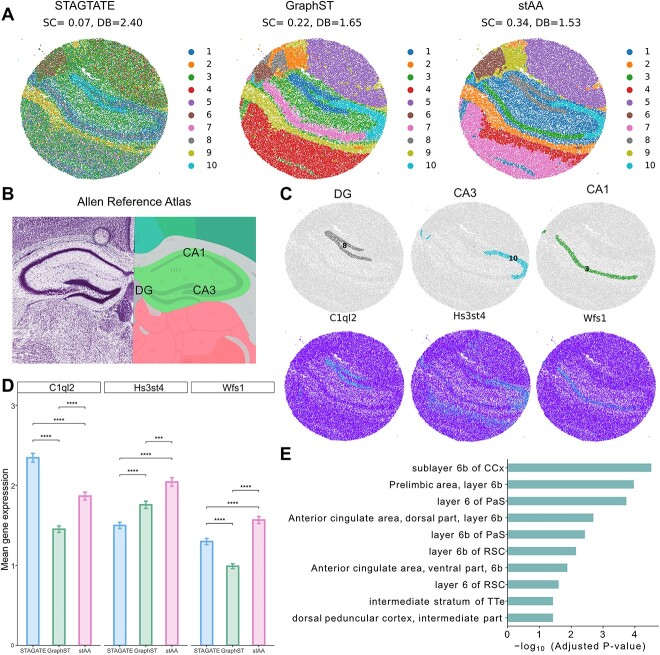
A comparison analysis of STAGATE, GraphST and stAA on Slide-seqV2-based mouse hippocampus data. (**A**) The identified spatial domains of three compared approaches. There are 10 regions in each method. A higher SC score and lower DB index denote a better clustering performance. (**B**) The laminar structure of mouse hippocampus is provided by the Allen Reference Atlas (Coronal Atlas). (**C**) Top, the domains clustered by stAA is shown on each spatial location. Bottom, the expression levels of corresponding domain-specific marker genes. The examined domains are CA1 cells, CA3 cells and dentate cells. (**D**) Bar plots display the mean gene expression of marker genes specific to CA1 cells, CA3 cells and DB cells inferred by comparative methods. Wilcoxon test, ^*^^*^^*^P < 0.001, ^*^^*^^*^^*^P < 0.0001. (**E**) The enriched Allen brain atlas terms in cluster 4 versus cluster 7.

Figure 3A illustrates the spatial domains identified by STAGATE, GraphST and stAA on the mouse hippocampus data. The hippocampus is composed of three main regions: the cornu ammonis 1 (CA1)/CA2 area, the cornu ammonis 3 (CA3) area and the dentate gyrus (DG), as shown in Figure 3B of the Allen Reference Atlas [40]. However, STAGATE cannot distinguish between CA1 and CA3, and cluster 3 covers the entire tissue, making it unable to reveal the hippocampal structure accurately. In contrast, stAA and GraphST accurately identified these three regions, but GraphST seems to identify a wider hippocampal structure [41]. Based on the quantized indices (SC and DB), stAA had the best clustering accuracy, with an SC score of 0.34 and a DB index of 1.53. Furthermore, we confirmed the cluster accuracy of stAA by examining the expression of three major hippocampal marker genes (Figure 3C). Cluster 8 is highly enriched in DG, and clusters 10 and 3 mainly localize in CA3 and CA1, respectively. We further quantified the clustering performance of the three methods by detecting the expression levels of marker genes in the three hippocampal structures (Figure 3D). We observed significant differences in the average expression levels of marker genes among the three methods. Hs3st4 and Wsf1 are marker genes for CA1 and CA3, respectively, and stAA had the highest average expression levels for both [42]. C1ql2 is a marker gene for the DG region [43], and its average expression level is highest in STAGATE, indicating that STAGATE’s identification of the boundaries of the DG is more accurate. However, it fails to distinguish between the CA1 and CA3 areas. The quantitative results again support that stAA identifies spatial domains more accurately than GraphST and STAGATE. Interestingly, stAA divided the telencephalon into two clusters, 4 and 7, compared with the comparison method. So, we identified the differentially expressed genes and performed functional annotation to characterize the distinct functions of cluster 4 and cluster 7. We found that cluster 4 expressed high levels of Rprm, Hs3st4, Trbc2 and Cplx3 (Supplementary Figure 4), both genes related to excitatory neurons [44]. Also, the Allen mouse atlas enrichment analysis [45] is shown that cluster 4 maybe is the cortical layer 6 (Figure 3E).

**Figure 4:**
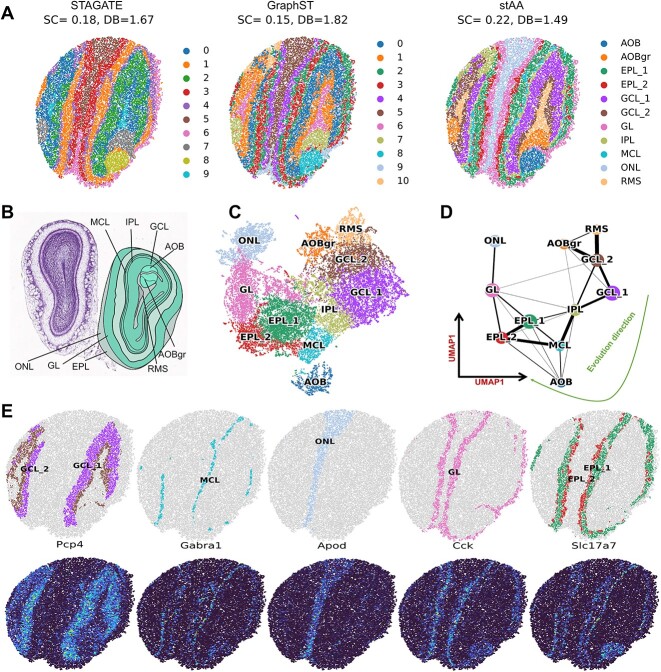
Clustering results of STAGATE, GraphST and stAA on the Slide-seqV2-based mouse olfactory bulb data. (**A**) The detected spatial regions of three compared techniques. There are 10 clusters in STAGATE and 11 clusters in GraphST and stAA. (**B**) The Allen Reference Atlas (Coronal Atlas) of mouse olfactory bulb data. (**C**) The UMAP plot of stAA’s clustering regions. (**D**) The spatial trajectory inference based on the UMAP plot. The evolution direction of these clusters in stAA is RMS, GCL, IPL, EPL and ONL. This trajectory is consistent with the spatial topological structure. (**E**) Representations of known marker genes for homologous regions of mouse olfactory bulb data (Pcp4: GCL, Gabra1: MCL, Apod: ONL, Cck: GL, Slc17a7: EPL).

stAA is compared with STAGATE and GraphST on the mouse olfactory bulb data (Figure 4). The reference atlas of this data is shown in Figure 4B, and the acronyms are explained in the Allen Reference Atlas (38). From the detected areas in Figure 4A, STAGATE has 10 clusters, and GraphST and stAA have 11 clusters. Based on the reference atlas, we renamed the identified clusters in stAA. By comparing with GraphST, the clusters in stAA have more evident and consistent borders with the Allen Reference Atlas. Furthermore, stAA achieved the highest SC score and smallest DB index, suggesting that stAA outperforms the other two comparison methods. The UMAP plot and inferred spatial trajectory are described in Figure 4C and D, and the developmental trajectory of stAA’s groups is consistent with the spatial topology structure of mouse olfactory bulb. The clustering performance of stAA is also validated using marker genes (Figure 4E). Pcp4 is shown to have high expressions in the identified GCL area, which is consistent with the results of *in situ* hybridization [46]. The mitral cell marker Gabra1 is highly expressed on the identified MCL cluster. Apod [47] is the marker gene of the ONL region, Cck [48] is significantly expressed in the GL group and Slc17a7 [49] is highly expressed in the EPL area. Based on the comparative analysis of these two mouse datasets, stAA was found to outperform the state-of-the-art clustering frameworks.

### stAA enables the recognition of mouse organogenesis atlas in the multiple samples profiled by Stereo-seq

To further illustrate the advantages of stAA, we compare it with conST, DeepST, STAGATE and GraphST on the mouse embryo data of E9.5 stage provided by Stereo-seq. There are five sections, including E1S1, E2S1, E2S2, E2S3 and E2S4. To see the overall performance on this E9.5 stage mouse embryo data, the average SC score and DB index of five compared methods are shown in Table 3. The clusters in stAA have high distinction and low similarity according to the mean values. The average SC score and DB index in stAA are 0.217 and 1.50, which implies that it has the best clustering performance on these datasets.

**Table 3:** SC score and DB index of five compared spatial clustering algorithm for mouse embryo dataset

Sections Methods	SC score						DB index					
	E1S1	E2S1	E2S2	E2S3	E2S4	Mean	E1S1	E2S1	E2S2	E2S3	E2S4	Mean
conST	0.19	0.19	0.20	0.12	0.21	0.19	1.64	1.54	1.51	1.89	1.64	1.63
DeepST	0.12	0.11	0.10	0.10	0.08	0.10	1.97	1.91	2.08	2.05	2.10	2.05
STAGATE	0.18	0.20	0.19	0.20	**0.23**	0.20	1.62	1.61	1.51	1.59	**1.34**	1.59
GraphST	0.19	0.13	0.14	0.12	0.18	0.14	1.58	1.68	1.88	1.90	1.61	1.68
stAA	**0.24**	**0.21**	**0.24**	**0.24**	0.16	**0.22**	**1.53**	**1.53**	**1.43**	**1.43**	1.60	**1.50**

Highest SC score for each dataset is in bold; Smallest DB index for each dataset is in bold.

To enhance the clarity of our spatial domain identification results, we have presented depictions of the spatial domains identified by each comparative method, alongside those identified by stAA for the E1S1 and E2S1 data. We have also included relevant marker gene information. For more comprehensive details, please refer to Supplementary Figures 5 and 6, as well as Note 4.

### stAA can delineate spatial heterogeneity of human breast cancer tissue

**Figure 5:**
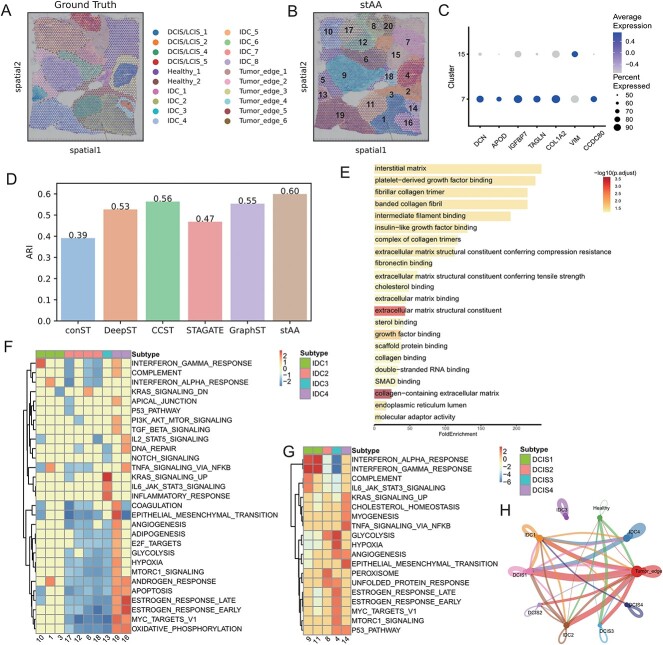
Results of spatial clustering using stAA on human breast cancer data and downstream analysis. (**A**) The manual annotation of this 10X Visium dataset is provided in the SEDR package. There are 20 segmented areas in this data and they are classified into four types. (**B**) The identified spatial domains using stAA. (**C**) Dot plot showing the differential expression genes of cluster 7 and cluster 15. (**D**) The ARI values of six compared methods. The ARI in conST is the lowest and stAA’s ARI is the highest. CCST is close to GraphST and they are better than DeepST and STAGATE. (**E**) The enriched GO terms in cluster 7 versus cluster 15. (**F**) Heatmap showing the enriched hallmark scores in each IDC cluster. (**G**) Heatmap showing the enriched hallmark scores in each DCIS/LCIS cluster. (**H**) Cell–cell interactions between subtypes, the link size represents the interaction strength.

To demonstrate the generalizability of stAA to cancer tissues, we evaluated its performance on human breast cancer data. This dataset is manually annotated in SEDR [15] package with 20 regions, which contain four morphotypes, DCIS/LCIS, IDC, tumor edge and healthy, as shown in Figure 5A. The 20 areas segmented by stAA are shown in Figure 5B. Many clusters are consistent with the manual annotation, such as 19, 13, 5, 9, 6 and 11. The clustering results of other methods are exhibited in Supplementary Figure 7. Interestingly, the Healthy_1 region is divided into two clusters in stAA (7 and 15) and GraphST (4 and 13) rather than one area in DeepST. Hence, we focus on analyzing the differential genes and biological functions of groups 7 and 15.

Several downstream analysis studies are conducted according to stAA’s clustering results. The differential expression of genes between clusters 7 and 15 is shown in Figure 5C. DCN, APOD, TAGLN and COL1A2 are highly expressed in cluster 7. These genes are related to the extracellular matrix. Cluster 15 is enriched with VIM, a marker of fibroblasts [50]. Supplementary Figure 8 describes the specific expression pattern of these genes in clusters 7 and 15. It is obvious that these genes (APOD, CCDC80, DCN, COL1A2, etc.) are enriched in cluster 7 rather than cluster 15. Furthermore, by conducting a Gene Ontology (GO) enrichment analysis [51], we find that cluster 7 is enriched with angiogenesis and extracellular matrix pathways, including ‘platelet-derived growth factor binding’, ‘extracellular matrix structural constituent’ and ‘collagen-containing extracellular matrix’ (Figure 5E). These results are also consistent with the cell types obtained using single-cell data deconvolution in previous studies [16, 24].

Using stAA’s identified domains, we could reclassify the DCIS/LCIS and IDC regions in the ground truth. Figure 5F illustrates the enriched hallmark scores of IDC domains (clusters 10, 1, 3, 17, 12, 6, 18, 13, 19 and 16) [52], which revealed that the function of each group is somewhat different. We divide these groups into four IDC subtypes based on their main function. IDC4 (clusters 19 and 16) has a high malignancy and is regulated by the hormone. IDC2 is an immunomodulatory suppression group, while IDC3 is driven by the KRAS mutation. Similarly, we reclassify the DCIS/LCIS regions according to their biological functions (Figure 5G). As a result, there are also four subtypes. For example, DCIS1 situates in the immune activation status. Inversely, while DCIS3 has the highest malignancy, DCIS2 locates in the intermediate state between DCIS1 and DCIS3. DCIS4 is driven by the mutation and has the epithelial mesenchymal transition function. Furthermore, we performed cell–cell communication analysis using CellChat [53] to gain a deeper insight into these subtypes. Consistent with the functional enrichment results, the immunologically activated subpopulations IDC1, IDC2 and IDIS1 exhibited more interactions with other domains. In contrast, the KRAS-mutated subpopulations IDC3 and DCIS4 showed little interaction with other domains (Figure 5H, Supplementary Figure 9). These results indicate that stAA can identify spatial domains with different functions that can be used for the analysis of tumor samples.

## DISCUSSION

Spatial clustering task is a fundamental and significant procedure to analyze the spatial transcriptomics data. In this paper, we propose a novel spatial clustering framework (stAA) by integrating the Wasserstein generative adversarial network and graph neural network. Three aspects of efforts have been conducted to improve the spatial clustering performance in stAA. Firstly, a variational graph autoencoder is used to dispose of the gene expression and spatial position information. Herein, the reconstruction loss could restrict the latent space of the encoder. Then, a classifier is constructed after the latent embedding generation. The cross-entropy loss in the classifier enables the latent embedding to better capture the structure of the gene expression profiles. Next, an adversarial training scheme is applied to further improve the quality of latent representation. The Wasserstein distance in this network helps to quantize the real and prior distributions, increasing separation between different cells and leading to better clustering performance. Finally, the derived cell embedding is employed to detect the spatial regions of different tissues and execute other downstream analyses.

To access stAA’s performance, we compared stAA with multiple state-of-the-art spatial clustering algorithms on different spatial transcriptomics datasets. Our experimental results proved that stAA is markedly superior to other state-of-the-art algorithms. First, we found that stAA outperformed other methods on the widely used human DLPFC dataset, achieving the largest ARI score and the best performance in most of the 12 samples. In addition, stAA was capable of identifying the fine structures of the mouse hippocampus and olfactory bulb, as well as significant spatial domains with specific functions in tumors. Moreover, by combining stAA-derived cell embeddings with clustering labels, downstream tasks such as trajectory inference and cell–cell communication analysis can be performed. Our experimental results showed that stAA accurately identified the spatial differentiation trajectories. In addition, we evaluated the performance of stAA on a single-cell resolution *in situ* hybridization-based dataset, which was generated using the STARMAP technology (see Supplementary Figure 10 and Note 5 for details).

In conclusion, stAA is a powerful and versatile model that can handle various spatial transcriptomics datasets and has an exceptional ability in gaining novel insights into spatial transcriptomic studies. However, stAA also has its limitations. For example, morphological images have the potential to improve the clustering accuracy, but how to incorporate them faces technological challenges. Moreover, the downstream clustering algorithm used in current frameworks is a traditional method, and replacing it with advanced techniques requires further exploration.

**Key Points**

- A novel adversarially variational graph embedding autoencoder is constructed for spatial domain identification study.
- stAA generates cell embedding by leveraging gene expression and spatial information using graph neural networks and enforces the distribution of cell embeddings to a prior distribution through Wasserstein distance. The adversarial training process can make cell embeddings better capture spatial domain information and more robust. Moreover, stAA incorporates global graph information into cell embeddings using labels generated by pre-clustering.
- stAA outperforms the state-of-the-art methods and achieves better clustering results across different profiling platforms and various resolutions. Also, stAA can identify fine-grained structures in tissues, recognize different functional subtypes within tumors and accurately identify developmental trajectories.

## Supplementary Material

Supplementary_bbad500

## Data Availability

The data used in this paper are all publicly available. Detailed information and publicly accessible source links can be found in the Supplementary Note 3.

## CODE AVAILABILITY

The stAA Python package is available at https://github.com/CSUBioGroup/stAA.

## Author Biographies

**Zhaoyu Fang** is with the School of Computer Science and Engineering at Central South University, Hunan, China. Her research interests include single-cell RNA-seq data analysis, deep learning and machine learning.

**Teng Liu** is with the Clinical Research Center (CRC), Medical Pathology Center (MPC), Cancer Early Detection and Treatment Center (CEDTC), Chongqing University Three Gorges Hospital, Chongqing University, Wanzhou, Chongqing, China. His research interests include single-cell RNA data analysis, spatial revolved transcriptomics, deep learning and deep reinforcement learning.

**Ruiqing Zheng** received the BS, MS and PhD degrees in Computer Science from Central South University, Changsha, China, in 2009, 2013 and 2021, respectively. He is currently a Lecturer at the School of Computer Science and Engineering, Central South University. His main research interests include bioinformatics and single-cell data analysis.

**Jin A** is a master at the School of Computer Science and Engineering, Central South University. Her current research interests include bioinformatics and single-cell data analysis.

**Mingzhu Yin** is with the Clinical Research Center (CRC), Medical Pathology Center (MPC), Cancer Early Detection and Treatment Center (CEDTC), Chongqing University Three Gorges Hospital, Chongqing University, Wanzhou, Chongqing, China, and also with Translational Medicine Research Center (TMRC), School of Medicine Chongqing University, Shapingba, Chongqing, China. His research interests include bioinformatics, single-cell and spatial omics, epigenome and non-coding RNA, and deep learning.

**Min Li** received her BS degree in Communication Engineering and MS and PhD degrees in Computer Science from Central South University, Changsha, China, in 2001, 2004 and 2008, respectively. She is currently a Professor at the School of Computer Science and Engineering, Central South University. Her main research interests include bioinformatics and system biology.
