# Supplementary material for: stAA: adversarial graph autoencoder for spatial clustering task of spatially resolved transcriptomics
Source: Brief Bioinform. 2024 Jan 6;25(1):bbad500. doi: 10.1093/bib/bbad500 (PMC10772985; doi:10.1093/bib/bbad500)
Supplement: Supplementary_bbad500 [file supplementary_bbad500.docx]

**Supplementary Materials For**

**stAA: Adversarial graph autoencoder for spatial clustering task of spatially resolved transcriptomics**

**Zhao-Yu Fang^1†^, Teng Liu^2,3†^, Ruiqing Zheng^1^, Jin A^1^, Ming-Zhu Yin^2,3*^, Min Li^1*^**

^1^School of Computer Science and Engineering, Central South University, Changsha, Hunan 410083, P.R. China

^2^Clinical Research Center (CRC), Medical Pathology Center (MPC), Cancer Early Detection and Treatment Center (CEDTC), Chongqing University Three Gorges Hospital, Chongqing University, Wanzhou, Chongqing, 404031, P.R. China

^3^Translational Medicine Research Center (TMRC), School of Medicine, Chongqing University, Shapingba, Chongqing, 401331, P.R. China.

*To whom correspondence should be addressed. Tel: +86-13975134596; Email: limin@mail.csu.edu.cn

Correspondence may also be addressed to Ming-zhu Yin Tel: +86-15873141982; Email: [yinmingzhu2008@126.com](mailto:yinmingzhu2008@126.com)

†The authors wish it to be known that, in their opinion, the first two authors should be regarded as Joint First Authors.

**Supplementary Figures**

**Supplementary Figure 1.** The boxplot of the variance of ARI values for seven spatial clustering approaches on the DLPFC data.

**Supplementary Figure 2.** Comparison of clustering accuracy on the DLPFC data.

**Supplementary Figure 3.** ARI comparison for stAA with different hidden layers in GNN across 12 samples in DLPFC data.

**Supplementary Figure 4.** Dot plot showing the differential expression genes of the stAA identified cluster 7 and cluster 4 on the human breast cancer data.

**Supplementary Figure 5.** The clustering areas of conST, DeepST, STAGATE, GraphST, and stAA on the mouse embryo data (Section E9_E1S1).

**Supplementary Figure 6.** The clustering areas of conST, DeepST, STAGATE, GraphST, and stAA on the mouse embryo data (Section E9_E2S1).

**Supplementary Figure 7.** Comparison of clustering accuracy on the human breast data.

**Supplementary Figure 8.** The marker gene expression of groups 7 and 15 in stAA results on the human breast data.

**Supplementary Figure 9.** Cell-cell interactions between subtypes on the human breast cancer data.

**Supplementary Figure 10.** Comparison of clustering accuracy on the STARmap data.

**Supplementary Notes**

**Supplementary Note 1.** Details in graph construction.

**Supplementary Note 2.** Hyperparameter Configuration of stAA.

**Supplementary Note 3.** Description of all datasets used in this study.

**Supplementary Note 4.** Results of spatial clustering using stAA on MOSTA dataset.

**Supplementary Note 5.** Results of spatial clustering using stAA on STARmap dataset.


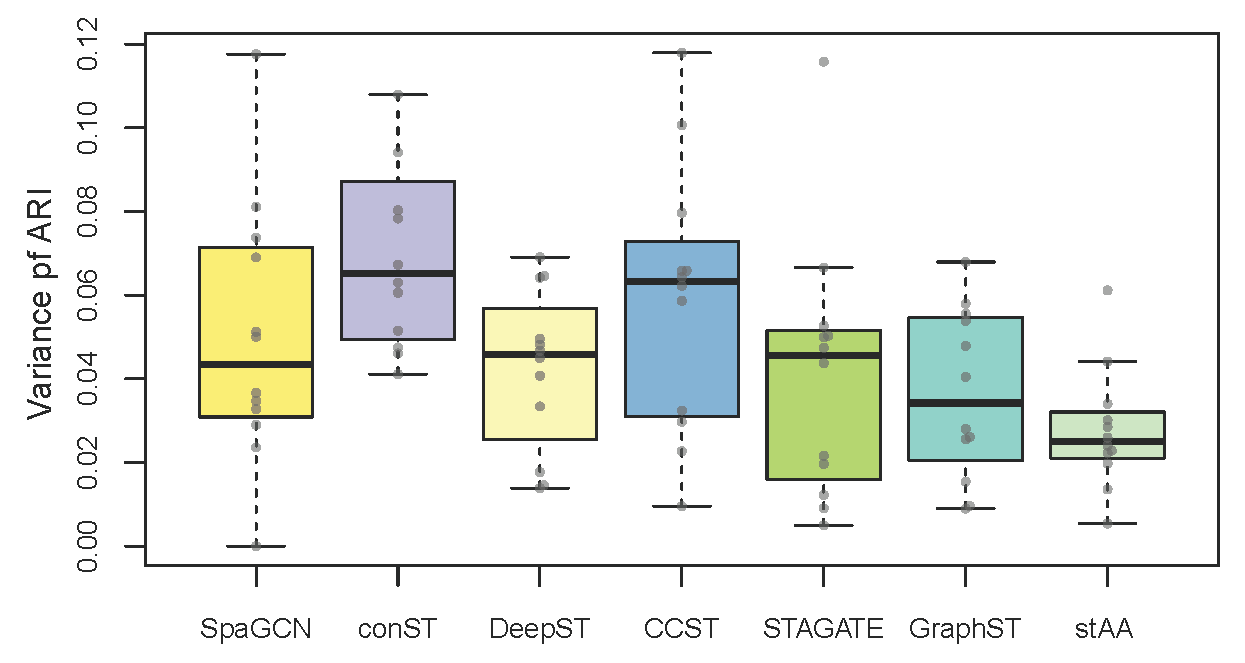


**Supplementary Figure 1.**The boxplot of the variance of ARI values for seven spatial clustering approaches. This variance is computed based on 12 samples in DLPFC data. Each sample is running 10 times to reduce the randomness. A higher variance denotes a bigger fluctuation. In these seven methods, conST and CCST have the higher ARI variances. The variances in SpaGCN, DeepST, STAGATE, and GraphST are close. The presented stAA has the lowest ARI variance.


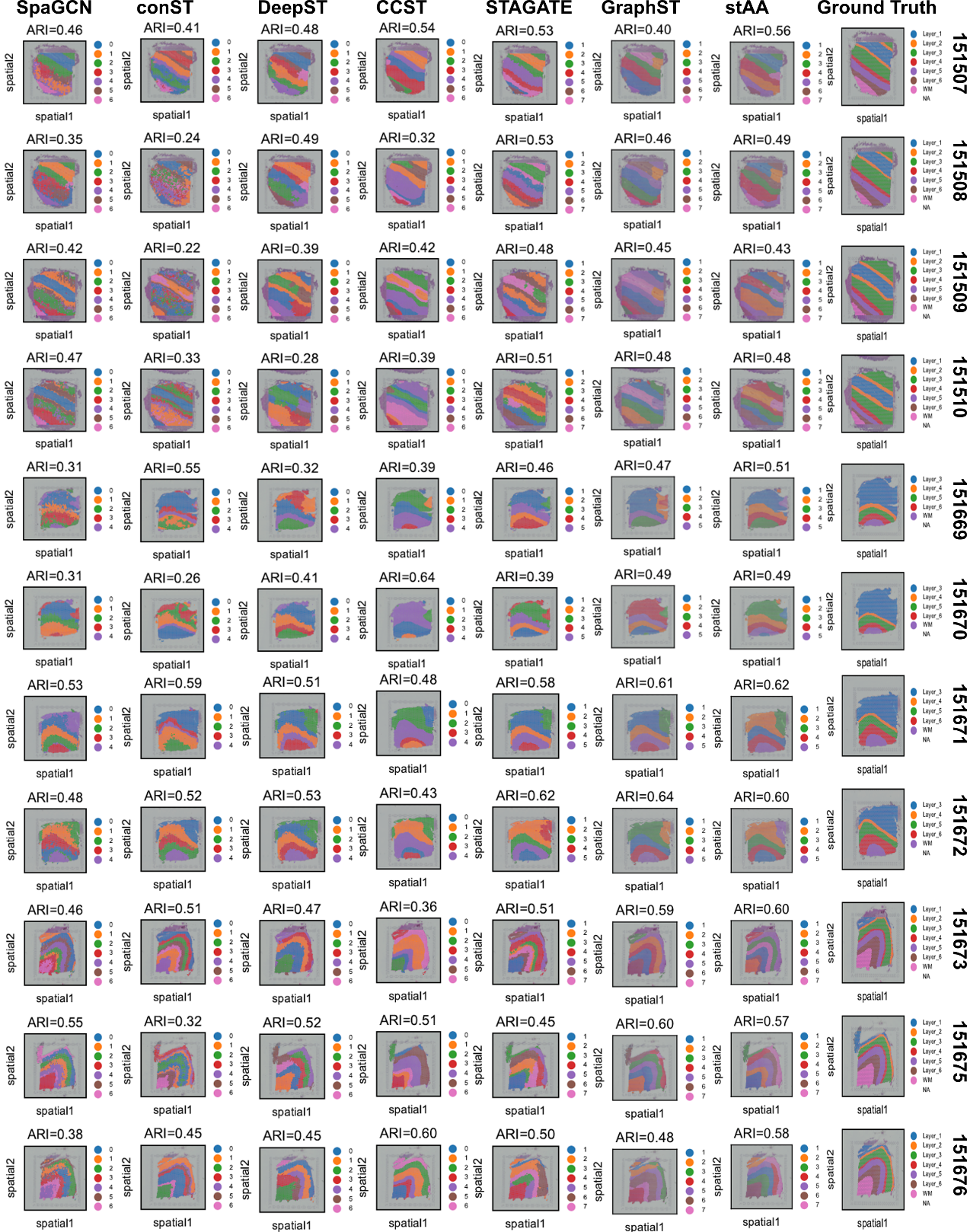


**Supplementary Figure 2.** Comparison of spatial domains by clustering assignments via SpaGCN, conST, DeepST, CCST, STAGATE, GraphST, stAA and ground truth for 11 samples besides sample 151674 of the DLPFC dataset. Each ARI score is derived using one method on one sample 10 times. Each row implies seven methods for one same sample. Each column indicates one method for 11 samples except sample 151674. The results of sample 151674 are displayed in Figure 2. By comparing these methods in each sample, the stAA has the highest ARI in most cases.


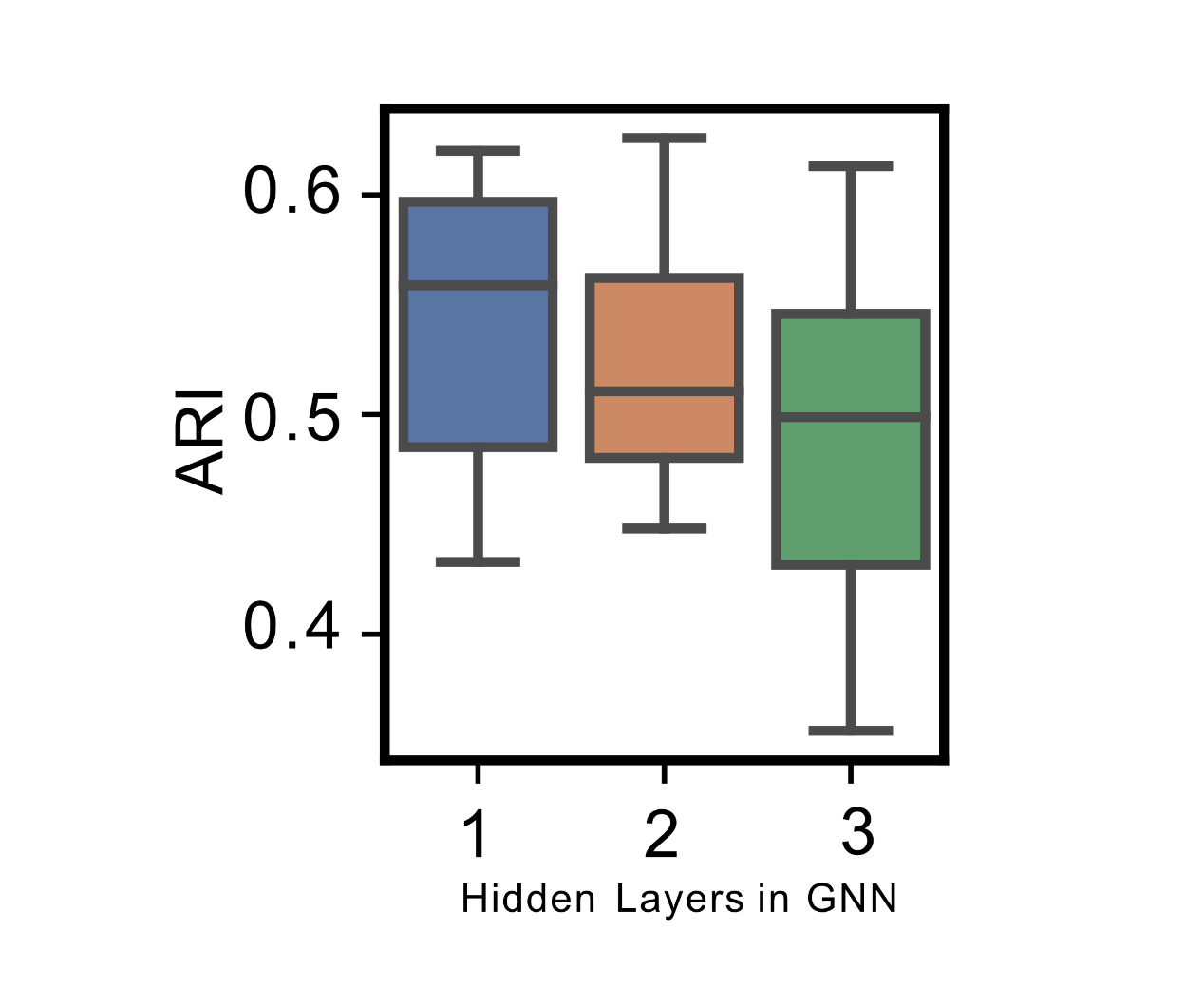


**Supplementary Figure 3.** ARI comparison for stAA with different hidden layers in GNN across 12 samples in DLPFC data.


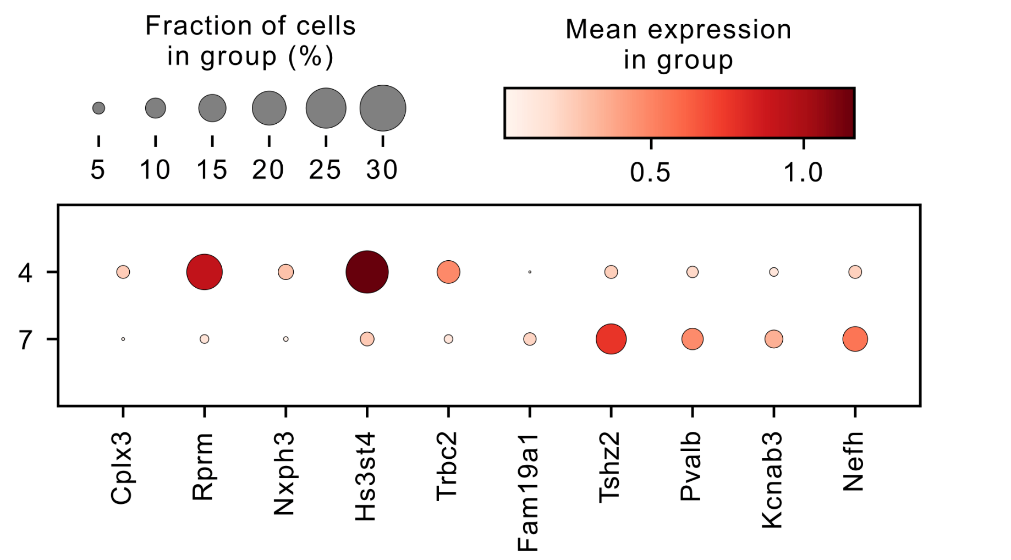


**Supplementary Figure 4.** Dot plot showing the differential expression genes of the stAA identified cluster 7 and cluster 4 on the human breast cancer data.


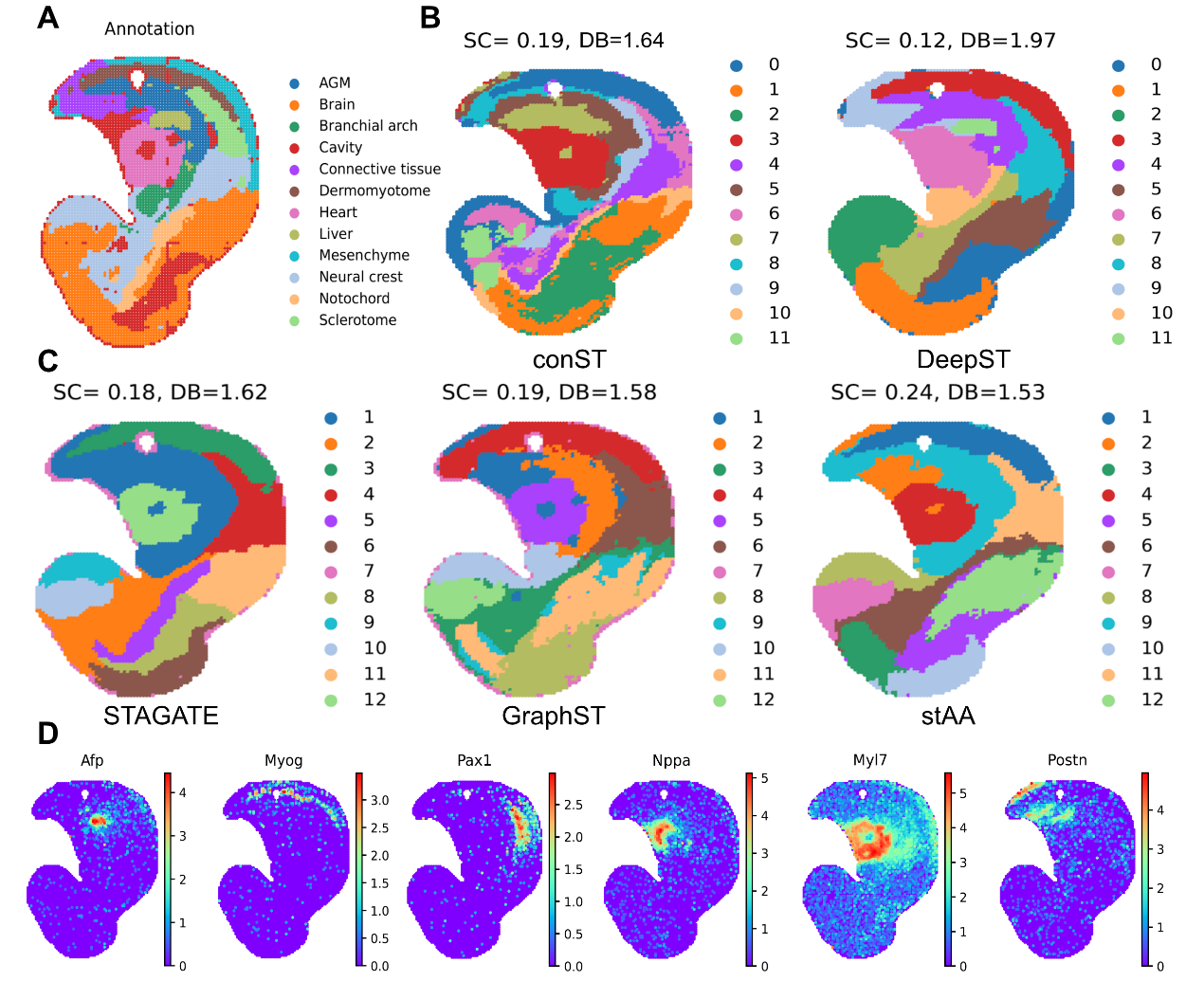


**Supplementary Figure 5.** Experimental results of five methods (conST, DeepST, STAGATE, GraphST, and stAA) on the mouse embryo data (Section E1S1). **A**. The manual annotation of mouse embryo data on 9 days stage. There are 12 regions in this data. **B**. The clustering results of conST and DeepST with their SC scores and DB indices. **C**. The identified clusters of STAGATE, GraphST, and stAA with the SC scores and DB indices. According to the evaluation criteria, stAA has the highest SC score and lowest DB index. It denotes that stAA has the best clustering performance. **D**. The marker genes of each organ in the mouse embryo. For example, My17, Postn, and Myog have significant expression in the heart, connective tissue, and dermomyotome.


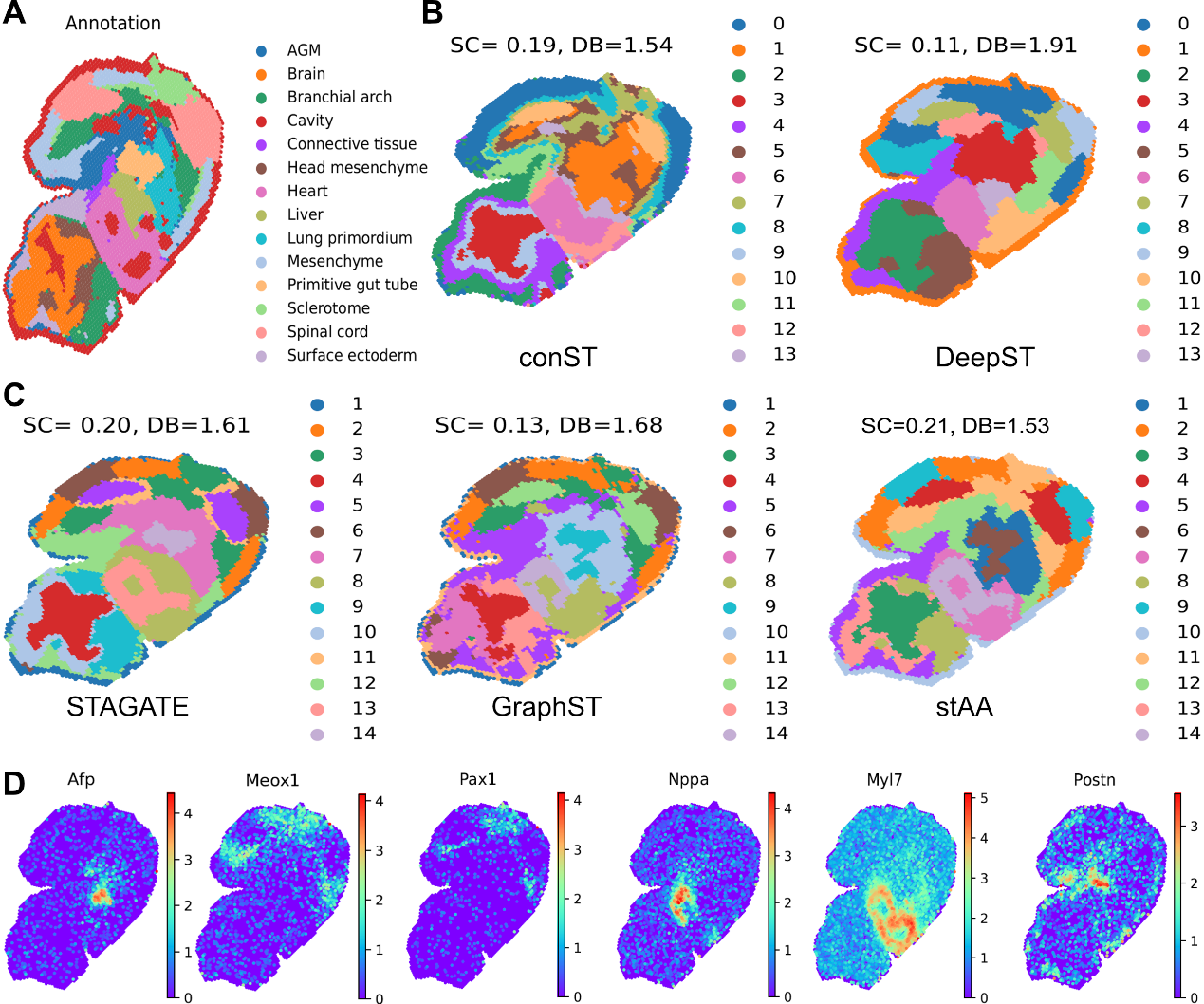


**Supplementary Figure 6.**The clustering areas of conST, DeepST, STAGATE, GraphST, and stAA on mouse embryo data (Section E9_E2S1). **A.** The ground truth of this mouse embryo data on 9 days stage. There are 14 domains in this data. **B.** The clustering results of conST and DeepST with their SC scores and DB indices. **C.** The detected areas in STAGATE, GraphST, and stAA. stAA also has the highest SC score and lowest DB index in this sample. **D.** The marked genes of different organs in mouse embryo, such as *MyI7*, *Afp*, and *Pax1*.


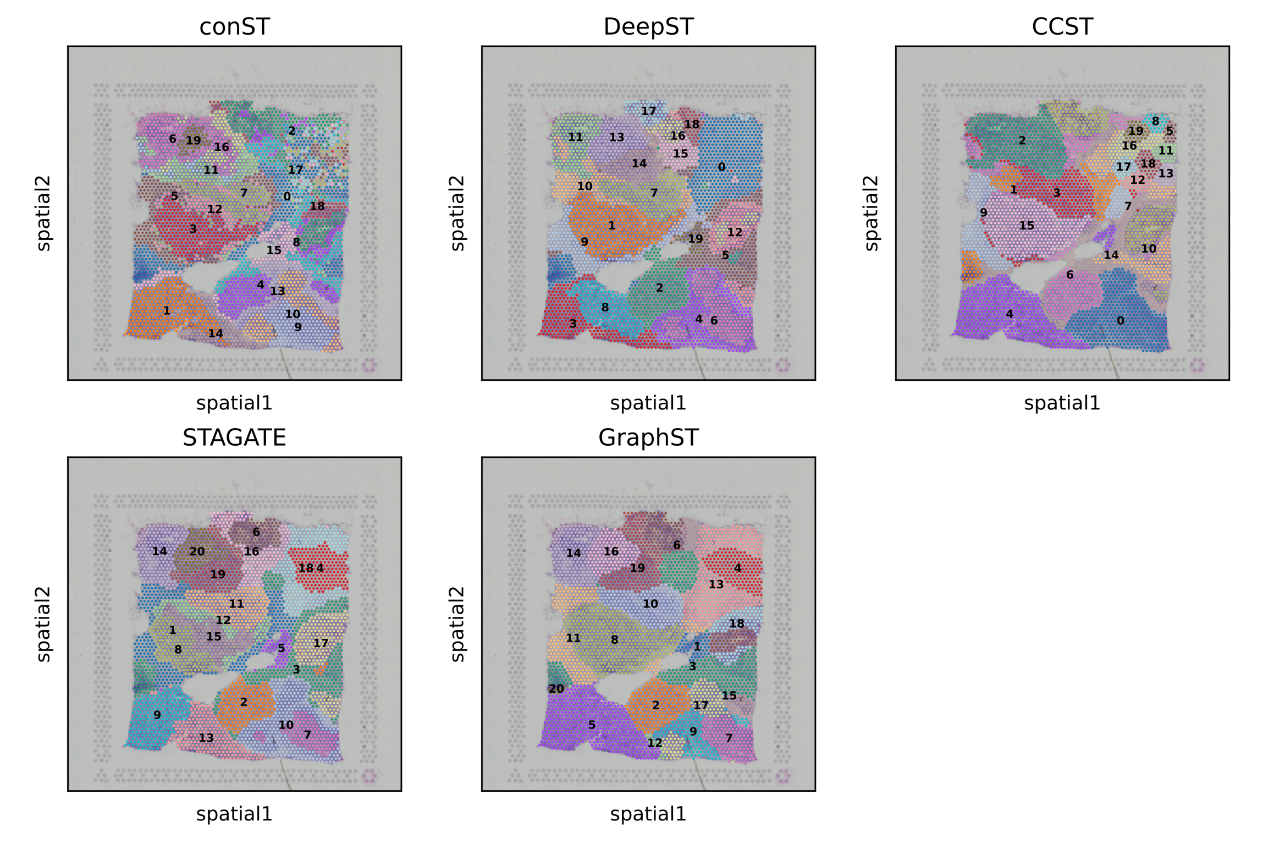


**Supplementary Figure 7.**The detected regions of benchmarking methods (conST, DeepST, CCST, STAGATE, and GraphST) on the human breast cancer data. The number of clusters is 20. The manual annotation of this data is provided in SEDR package.


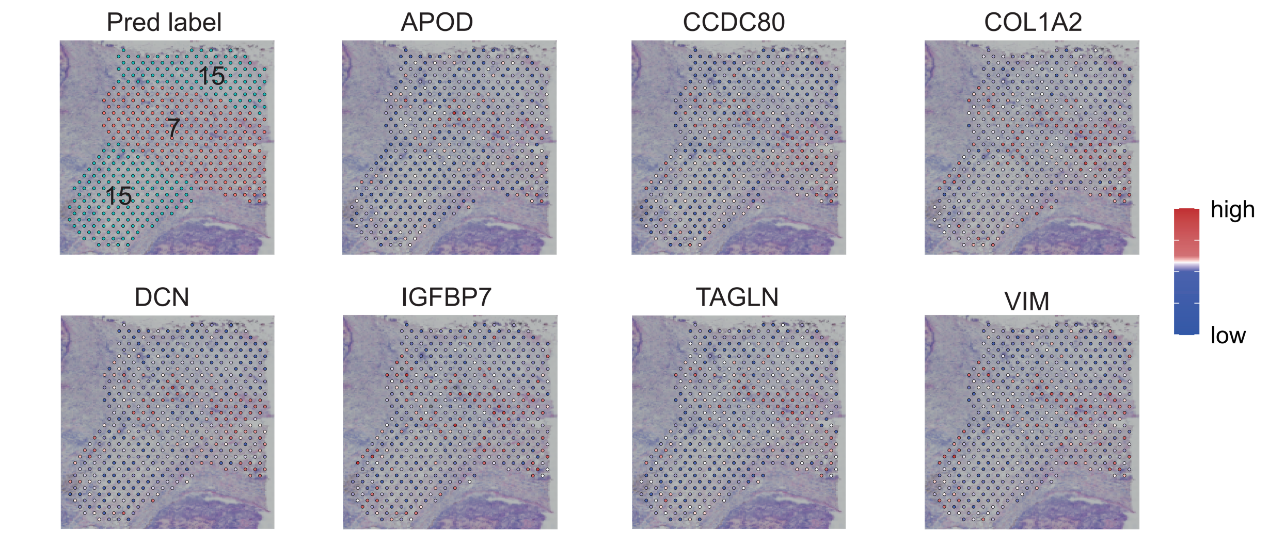


**Supplementary Figure 8.**The marker gene expression of groups 7 and 15 in stAA results on the human breast cancer data. The first subfigure describes the prediction labels of clusters 7 and 15 in stAA. The remaining subfigures illustrate the differential genes between groups 7 and 15.


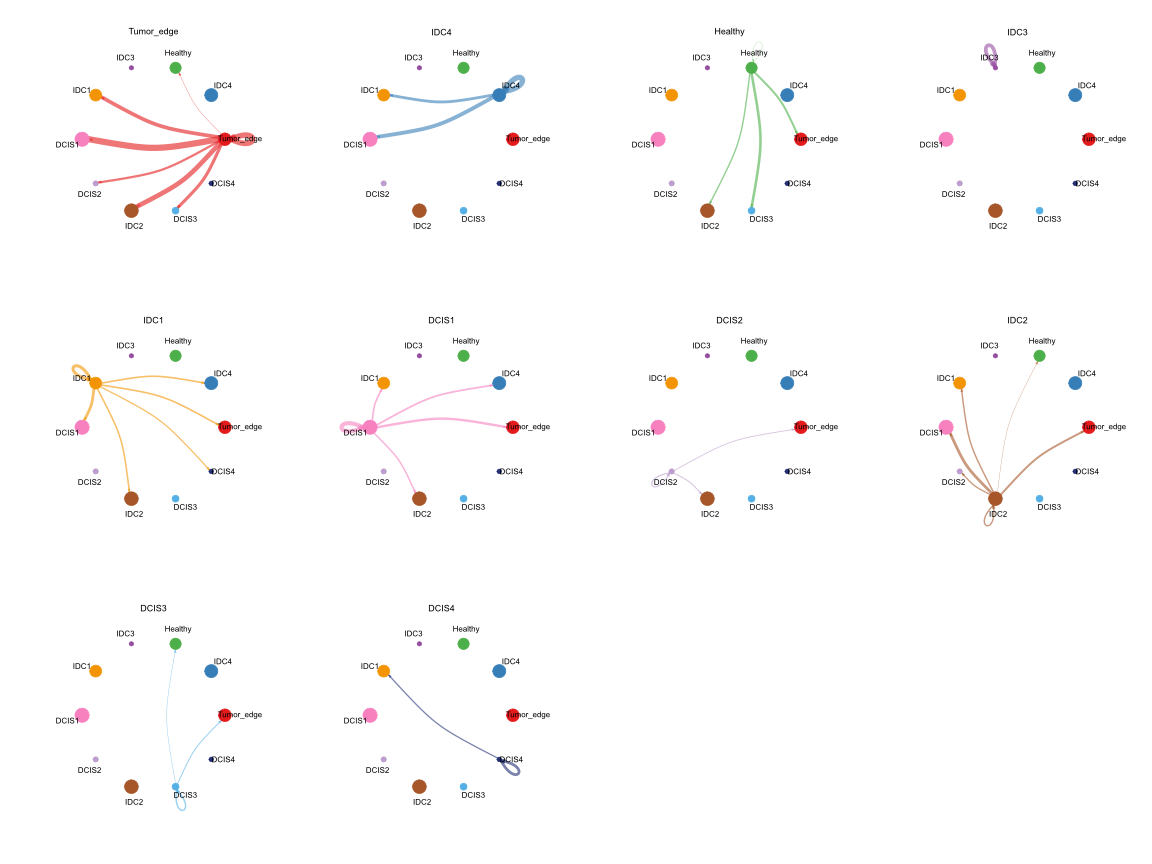


**Supplementary Figure 9.** Cell-cell interactions between subtypes on the human breast cancer data, the link size represents the interaction strengthen. Each subplot represents the cell-cell communication patterns of a specific cell type (the cell type name is displayed as the subplot title).


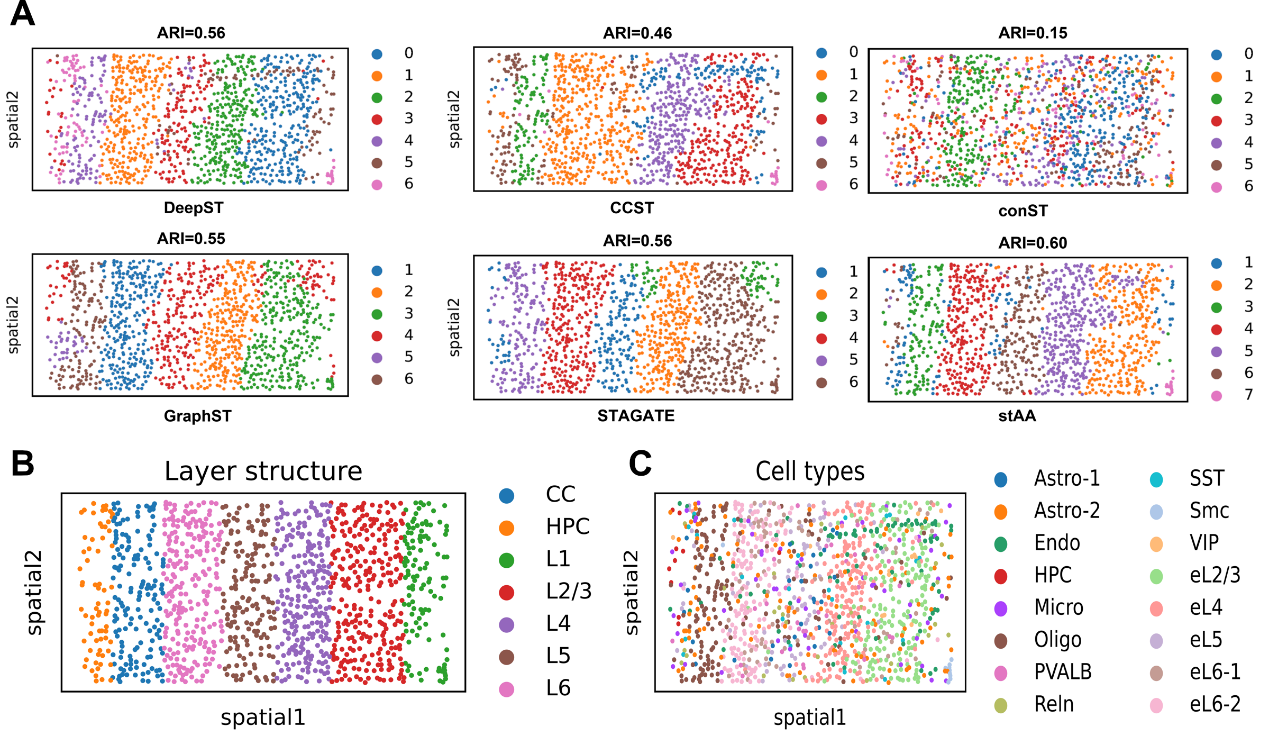


**Supplementary Figure 10.**Compared clustering layers of six methods on the STARmap dataset. **A.** The identified layers of DeepST, CCST, conST, GraphST, STAGATE, and stAA. The ARI values are calculated based on the prediction labels of each approach and the ground truth. stAA has the biggest ARI score, 0.60. **B.** The ground truth of this STARmap data, the distinction boundaries of each layer are clear. **C.** The cell types in this STARmap data. There are 16 cell types in this dataset and they locate into seven layers.

**Supplementary Note 1. Details in graph construction**

To fully utilize spatial position information, an graph $G$ is constructed where each spot represents a node, and pairs of cells located in close proximity to each other in space are connected. The Euclidean distance between spots is first calculated, and a predefined radius is used to construct the adjacency matrix for most of the data, ensuring that each spot contains an average of 5-15 neighbors, as suggested by previous studies [1-2]. The specific radius used is shown in the following Supplementary Table 1. For the MOSTA data, the nearest 5 neighbors were used for graph construction.

Supplementary Table 1. Hyperparameter of graph mode

| Datasets | DLPFC | Human breast cancer | Mouse olfactory bulb | Mouse hippocampus | STARmap |
| --- | --- | --- | --- | --- | --- |
| radius | 150 | 300 | 50 | 40 | 400 |

**Supplementary Note 2. Hyperparameter Configuration of stAA**

The calculative course of the presented stAA is conducted based on PyTorch_pyG [3] in Python. PyTorch_pyG is a library consists of many realized GNN algorithms for structured data. For the VGAE module in stAA, 3000 highly variable genes are selected to constitute the feature matrix, the particular GNN in the autoencoder is SGConv [4], the dimensions of hidden layer and latent embedding space are 256 and 128, respectively. The weights of the BEC loss and CE loss are 0.4 and 0.6 in the VGAE model. These loss functions are optimized by an Adam optimizer [5] with an initial learning rate of 1e-5 and weight decay of 5e-05. The activation function of GNN is rectified linear unit (ReLU). The number of epochs is 1000 by default and the number of GNN layers is 2.

For the WGAN model, the two-layer hidden dimensions are 64 and 32, and its learning rate is also 1e-5. The activation function in regularizer and classifier is Sigmoid. The Wasserstein distance is calculated with parameters in the Wasserstein regularizer clamped into [-0.01, 0.01]. And for the loss function , the $\lambda$ is set to 0.4.

The mouse olfactory bulb dataset used the Louvain [6] algorithm for downstream clustering in comparison methods (GraphST [1] and STAGATE [11]). To ensure fairness, the stAA and comparison methods also used the Louvain algorithm (with resolution=0.5) for downstream clustering in the mouse olfactory bulb dataset. In contrast, the mclust [7] method was used for clustering in all other datasets.

In this paper, the experiments are conducted on Ubuntu 18.04.6 LTS with Intel (R) Core (TM) i9-10980XE CPU @ 3.00 GHz and 64 GB memory. We use GPU to accelerate the training process of GNN. The version of GPU is GeForce RTX™ 3090Ti.

**Supplementary Note 3. Description of all datasets used in this study**

*10X Visium data.* One spot in 10X Visium technology contains 5 to 10 cells and its sequencing depth is relatively high. Human brain dataset is a fashionable spatial transcriptome dataset in the spatial clustering task. This dataset is released on the 10x Genomics Visium platform and well annotated by the spatialLIBD project [8]. It concentrates on the human dorsolateral pre-frontal cortex (DLPFC) area, spanning six or four neuronal layers plus white matter for three subjects. There are 12 samples in this DLPFC data. Five or seven domains are manually labelled on these samples, and each region has clear boundary. These layers have distinct chronological order; thus, this dataset is also used for spatial trajectory inference.

The second spatial dataset is derived from human breast cancer, also a 10X Visium spatial transcriptomic data. This tumor sample has high heterogeneity and complicated microenvironments. The authors in SEDR [9] manually labelled this data relying on the morphological image and gene expression profiling. 20 domains are recognized in this dataset, including four morphotypes, which are ductal carcinoma in situ/lobular carcinoma in situ (DCIS/LCIS), invasive ductal carcinoma (IDC), tumor edge areas, and healthy regions. These annotated cell types could also be regarded as the golden standard for clustering evaluation. The number of spots and genes is about 3700 and 36000. The sequencing depth in this data is 150392 reads per spot. The median genes and median UMI counts are 6026 and 20762, respectively.

*Slide-seqV2 data.* The mouse hippocampus data based on the Slide-seqV2 technology is discussed in this paper. Slide-seqV2 [10] is the upgraded version of the original Slide-seq technique, wherein the localized mRNAs capture efficiency is improved. One spot in Slide-seqV2 has near-cellular resolution. The pre-processed data of the mouse hippocampus is available in the Squidpy [11] package. Herein, there are 14 cell types in this data. The spots and sequenced genes are about 41000 and 4000. In this paper, we use Allen Reference Atlas and mouse brain marker genes to evaluate the clustering performance on this data.

The second Slide-seqV2-based data is mouse olfactory bulb dataset. The comparison analysis is achieved on one sample termed Puck_200127_15 of mouse olfactory bulb data. This dataset has been pre-processed and released through the STAGATE approach [12], and it consists of approximately 20,000 spots and 11,000 genes. It is obvious that the number of spots increases sharply when compared with the 10X Visium data. It is attributed to near-cellular resolution of the Slide-seqV2 data. To evaluate the performance of the stAA, cell types are annotated based on the Allen Reference Atlas and mouse brain gene expression atlas [13]. This annotation is considered the ground truth to assess clustering results. Marker genes for the mouse hippocampus and olfactory bulb regions are well-known and can be obtained from the Allen mouse brain atlas and Slide-seqV2 paper. The clustering results obtained should match the pattern of these marker genes to indicate good performance.

*Stereo-seq data.* Stereo-seq is a DNB-based genome-wide technology that combines high sensitivity, single-cell resolution, and a large field of view [14]. It is reported to capture 133776 UMI counts per 100 μm (diameter) bin. This is superior to 10X Visium and Slide-seqV2 data. The data profiled by Stereo-seq is the mouse organogenesis spatiotemporal transcriptomic atlas (MOSTA) database. This database focuses on sagittal sections from C57BL/6 mouse embryos. There are eight developmental stages of mouse embryos from E9.5 to E16.5. In this paper, we discuss spatial clustering task on E9.5 stage that include five replicate samples. The sequencing depth of this dataset is close to that of the mouse olfactory bulb sample.

*STARmap data.* STARmap utilizes combinatorial barcoding and imaging to detect transcriptome-wide expression patterns at single-cell resolution in situ [15]. We used a dataset from mouse visual cortex that exhibits distinct seven layers. This dataset covers expression information for approximately 1000 genes and offers insights into the distribution of different neuronal subtypes. The presented stAA and benchmarking methods are estimated on the above datasets and the clustering performance is evaluated.

The experiment datasets used in this article are available at the following links. 1) DPLFC: The primary source: https://github.com/LieberInstitute/spatialLIBD; The processed version: https://www.nature.com/articles/s41593-020-00787-0. 2) Human breast cancer: The primary source: https://www.10xgenomics.com/resources/datasets/human-breast-cancer-block-a-section-1-1-standard-1-1-0; The processed version: https://github.com/JinmiaoChenLab/SEDR_analyses/. 3) Slide-seqV2 mouse olfactory bulb: The primary source: https://singlecell.broadinstitute.org/single_cell/study/SCP815/highly-sensitive-spatial-transcriptomics-at-near-cellular-resolution-with-slide-seqv2#study-summary; The processed version: https://stagate.readthedocs.io/en/latest/T3_Slide-seqV2.html. 4) Slide-seqV2 mouse hippocampus: The primary source: https://singlecell.broadinstitute.org/single_cell/study/SCP354/slide-seq-study; The processed version: https://squidpy.readthedocs.io/en/stable/api/squidpy.datasets.slideseqv2.html. 5) MOSTA database: The primary source: https://db.cngb.org/stomics/mosta/; The processed version: https://db.cngb.org/stomics/mosta/download/. 6) The mouse visual cortex STARmap data36 is accessible on https://www.dropbox.com/sh/f7ebheru1lbz91s/AADm6D54GSEFXB1feRy6OSASa/visual_1020/20180505_BY3_1kgenes?dl=0&subfolder_nav_tracking=1. 7) Allen Reference Atlas: The primary source: https://mouse.brain-map.org/static/atlas; The processed version: <https://mouse.brain-map.org/experiment/thumbnails/100048576?image_type=atlas>.

**Supplementary Note 4. Results of spatial clustering using stAA on MOSTA dataset.**

The experimental results of the E1S1 section are presented in Supplementary Figure 4. The annotation of the E1S1 section of the mouse embryo dataset is shown in Supplementary Figure 4A, with 12 clusters representing different organs such as the brain, heart, liver, and neural crest. The clustering results of conST and DeepST are displayed in Supplementary Figure 4B, while Figure Supplementary Figure 4C shows the identified spatial domains of STAGATE, GraphST, and stAA, with 12 regions in each clustering framework. Both conST and GraphST are based on contrastive learning mechanisms, and their performance is close. By comparing the SC scores and DB indices, stAA has the best clustering accuracy and DeepST has the worst performance. To validate the stAA’s clustering groups, marker genes for different organs are highlighted in Supplementary Figure 4D. For example, MyI7 and Nppa is the biomarker of the heart [16] and is corresponding to cluster 4 in stAA. Cluster 2 in stAA denotes Connective tissue in mouse embryos, and Postn has a high expression in this area [17]. Pax1 is the marker gene of Sclerotome [18], which complies with cluster 11 in stAA (Supplementary Figure 4C).

The analogous comparison analysis of section E2S1 at E9.5 stage of this data is shown in Supplementary Figure 5. There are 14 clusters in the manual annotation, as displayed in Supplementary Figure 5A. The identified spatial areas of each approach are exhibited in Supplementary Figures 5B and 5C. DeepST and stAA are the worst and best techniques in these compared clustering frameworks. stAA is also close to the ground truth in this case wherein its SC score and DB index are 0.21 and 1.53, respectively. We also highlight the marker genes in this section, such as MyI7, Afp, and Pax1.

**Supplementary Note 5. Results of spatial clustering using stAA on STARmap dataset.**

The STARmap data has 16 cell types that locate in seven layers, which are described in Supplementary Figures 9B and 9C. The compared clustering results of six approaches on STARmap data are shown in Supplementary Figure 9. Based on the separated layer structure, the ARI value of each method is computed. stAA has the highest ARI (0.60) and is close to the ground truth. The borders of each layer in stAA are clear. conST has the worst clustering performance. The accuracy of DeepST, GraphST, and STAGATE is close and their ARI values are larger than 0.50. GraphST and STAGATE can only detect six layers, and the remaining methods can find seven layers. The experimental results demonstrate that stAA could handle datasets with different resolutions from different sequencing platforms and outperforms existing spatial clustering frameworks.

**Reference**

1. Long, Y., Ang, K.S., Li, M., Chong, K.L.K., Sethi, R., Zhong, C., Xu, H., Ong, Z., Sachaphibulkij, K. and Chen, A. (2023) Spatially informed clustering, integration, and deconvolution of spatial transcriptomics with GraphST. Nature Communications, 14, 1155.

2. Dong, K. and Zhang, S. (2022) Deciphering spatial domains from spatially resolved transcriptomics with an adaptive graph attention auto-encoder. Nat Commun, 13, 1739.

3. Fey, M., Lenssen, J. E. Fast graph representation learning with PyTorch Geometric. arXiv preprint. 2019;arXiv:1903.02428: doi: https://arxiv.org/abs/1903.02428.

4. Li, Y., Cai, T., Zhang, Y., Chen, D., Dey, D. What Makes Convolutional Models Great on Long Sequence Modeling?. arXiv preprint. 2022;arXiv:2210.09298: doi: https://arxiv.org/abs/2210.09298.

5. Zhang, Z. Improved adam optimizer for deep neural networks. In 2018 IEEE/ACM 26th international symposium on quality of service (IWQoS). 2018:1-2.

6. Blondel, V.D., Guillaume, J.-L., Lambiotte, R. and Lefebvre, E. (2008) Fast unfolding of communities in large networks. J. Stat. Mech: Theory Exp., 2008, P10008.

7. Scrucca, L., Fop, M., Murphy, T.B. and Raftery, A.E. (2016) mclust 5: clustering, classification and density estimation using Gaussian finite mixture models. The R journal, 8, 289.

8. Pardo, B., Spangler, A., Weber, L.M., Page, S.C., Hicks, S.C., Jaffe, A.E., Martinowich, K., Maynard, K.R. and Collado-Torres, L. (2022) spatialLIBD: an R/Bioconductor package to visualize spatially-resolved transcriptomics data. BMC genomics, 23, 434.

9. Fu, H., Xu, H., Chong, K., Li, M., Ang, K.S., Lee, H.K., Ling, J., Chen, A., Shao, L. and Liu, L. (2021) Unsupervised spatially embedded deep representation of spatial transcriptomics. Biorxiv, 2021.2006. 2015.448542.

10. Stickels, R.R., Murray, E., Kumar, P., Li, J., Marshall, J.L., Di Bella, D.J., Arlotta, P., Macosko, E.Z. and Chen, F. (2021) Highly sensitive spatial transcriptomics at near-cellular resolution with Slide-seqV2. Nature biotechnology, 39, 313-319.

11. Dong, K. and Zhang, S. (2022) Deciphering spatial domains from spatially resolved transcriptomics with an adaptive graph attention auto-encoder. Nat Commun, 13, 1739.

12. Palla, G., Spitzer, H., Klein, M., Fischer, D., Schaar, A.C., Kuemmerle, L.B., Rybakov, S., Ibarra, I.L., Holmberg, O. and Virshup, I. (2022) Squidpy: a scalable framework for spatial omics analysis. Nature methods, 19, 171-178.

13. Chon, U., Vanselow, D.J., Cheng, K.C. and Kim, Y. (2019) Enhanced and unified anatomical labeling for a common mouse brain atlas. Nature communications, 10, 5067.

14. Chen, A., Liao, S., Cheng, M., Ma, K., Wu, L., Lai, Y., Qiu, X., Yang, J., Xu, J. and Hao, S. (2022) Spatiotemporal transcriptomic atlas of mouse organogenesis using DNA nanoball-patterned arrays. Cell, 185, 1777-1792. e1721.

15. Wang, X., Allen, W.E., Wright, M.A., Sylwestrak, E.L., Samusik, N., Vesuna, S., Evans, K., Liu, C., Ramakrishnan, C. and Liu, J. (2018) Three-dimensional intact-tissue sequencing of single-cell transcriptional states. Science, 361, eaat5691.

16. Christoffels VM, Habets PE, Franco D et al. Chamber formation and morphogenesis in the developing mammalian heart, Developmental Biology 2000;223:266-278.

17. Hamilton DW. Functional role of periostin in development and wound repair: implications for connective tissue disease, Journal of Cell Communication and Signaling 2008;2:9-17.

18. Ebensperger C, Wilting J, Brand-Saberi B et al. Pax-1, a regulator of sclerotome development is induced by notochord and floor plate signals in avian embryos, Anatomy and embryology 1995;191:297-310.
